# Value-Based Healthcare and Enhanced Recovery After Surgery Implementation in a High-Volume Bariatric Center in Italy

**DOI:** 10.1007/s11695-020-04464-w

**Published:** 2020-02-24

**Authors:** Giulia Goretti, Giuseppe M. Marinari, Elena Vanni, Chiara Ferrari

**Affiliations:** 1 Quality Department, Humanitas Clinical and Research Center –IRCCS, via Manzoni, 56 20089 Rozzano, MI Italy; 2 Bariatric Surgery, Humanitas Clinical and Research Center –IRCCS, via Manzoni, 56 20089 Rozzano, MI Italy; 3 Performance audit, Humanitas Clinical and Research Center –IRCCS, via Manzoni, 56 20089 Rozzano, MI Italy; 4 Anesthesia, Humanitas Clinical and Research Center –IRCCS, via Manzoni, 56 20089 Rozzano, MI Italy

**Keywords:** Bariatric surgery, Value-based healthcare, Patient-centered care, Patient wellness, Performance measures, Quality improvement

## Abstract

**Background:**

Bariatric surgery is the most effective treatment for patients affected by morbid obesity. The Enhanced Recovery After Surgery (ERAS) protocol increases clinical outcomes, but the most recent literature shows incomplete patients’ adherence. This study aims to demonstrate the feasibility of applying a Value-Based Healthcare (VBHC) strategy associated with ERAS to increase patients’ engagement and outcomes.

**Method:**

A multiprofessional team redesigned the process considering ERAS recommendations and patients’ feedbacks. Outcomes that matter to patients were defined with structured patients’ interviews and collected in the electronic clinical record. Adherence to the pathway and the cost of the cycle of care were measured to demonstrate sustainability. A model was developed to grant its replicability.

**Results:**

A total of 2.122 patients were included. The lowest adherence to the protocol for a single item was 82%. 74% of excess weight loss; 90% better comorbidities control; 77.5% had no pain after surgery; 61% no postoperative nausea and vomiting. Zero mortality; 1.8% overall morbidity; 0.4% readmission and reoperation rate within 30 days. The average length of stay is 2.1 days. Patient-Reported Outcome Measures (PROMs) documented increased productivity and quality of life.

**Conclusion:**

Building a caring relationship by a multidisciplinary team, adding patient wellness in a VBHC framework on top of ERAS as a patient-centered approach, increases patients’ engagement and adherence to the pathway of care, resulting in better health outcomes (clinical and PROMs). The Value-Based Model is sustainable and replicable; it represents the prototype for redesigning other pathways and may become a model for other organizations.

## Introduction

Morbid obesity is an increasingly prevalent condition worldwide. It is a chronic disease associated with long-term comorbidities (i.e., diabetes, cardiac and respiratory diseases, and malignant neoplasms), reduced overall survival (higher any cause-any age mortality), mental illness (i.e., depression and anxiety), and poorer quality of life [1].

Morbid obesity has become a serious international public health concern with a considerable impact on both direct (i.e., drugs) and indirect costs (productivity losses due to working days lost and reduced productivity at work) [2, 3]. Indeed, the direct healthcare cost for obese adults is 42% higher than that for healthy adults [4]. Moreover, the estimated obesity-related medical cost in the USA is $147 billion/year on the top of indirect costs, related to morbidity, mortality, and productivity loss [2]. For instance, job absenteeism alone costs approximately $4.3 billion per year in the USA [5].

Bariatric surgery is currently the most effective treatment for morbid obesity, resulting in both sustained weight loss and improvement of obesity-related comorbidities [6]. An increasing number of bariatric procedures have been performed worldwide during the last 20 years and much effort has been made to identify efficient clinical management strategies for these patients.

The Enhanced Recovery After Surgery (ERAS) program is a clinical management approach designed to improve perioperative outcomes and decrease patient’s length of hospital stay (LOS), leading to reduced healthcare costs [7, 8]. Worldwide recommendations for an ERAS approach to bariatric patients are still under implementation although preliminary positive results [9].

Clinical evidence [10–12] reported a substantial issue concerning patients’ adherence to ERAS protocol. That is a relevant topic considering the relationship between adherence and survival [13] and outcome [12].

Value-Based Health Care (VBHC) is a strategic management framework that maximizes the ratio between health outcomes and costs [14–18]. Developed in 2006, it is based on three acting principles: (i) building value for the patients; (ii) basing the organization of medical practice on medical conditions and care cycles; (iii) measuring outcomes and economic costs [19]. To implement VBHC within the organizations, there is a 6-step approach [14] (Appendix 1). Case studies have been published, mainly in northern Europe, to describe the method and potential benefits in the application of the approach [19–25]. Up to this date, no studies have been performed in Italy and for obese patients worldwide to our knowledge.

Starting from ERAS, we redesigned the organizational pathway with the involvement of a multi-professional team, patients and their families. We defined and measured the patient-relevant outcomes and we compared them to the costs, as the strategy that VBHC suggests.

In this observational study, we show how to implement a VBHC strategy, starting from ERAS principles applied to a high volume bariatric center and the feasibility of this approach to achieve excellent clinical outcomes and better quality of life without increasing costs, through the involvement and engagement of patients.

The aim of this work is to increase patients’ adherence to their own cycle of care, to show the feasibility of this approach and to develop a replicable model.

## Methods

In July 2015, the bariatric surgery unit started its activity at a private and highly specialized academic hospital in northern Italy with strong assets and costs management and culture of measurement [26].

The bariatric team was already used to work combining clinical excellence and efficiency and brought its previous experience in Fast Track protocol; thinking about patient value, though, required a wider perspective.

For this reason, a multi-professional team (surgeon, anesthesiologist, lean manager, asset manager, scrub, pain and ward nurse, nutritionist, and psychologist) was organized to compose an integrated practice unit (IPU). Patients were interviewed by clinicians to collect their experiences and suggestions to improve their pathway of care. From July to October 2015 the team reached a consensus on the ERAS elements reported in Table 1. From November to December 2015, the team designed the process, and according to Lean principles (Appendix 2), the IPU defined the value for patients and clinicians.

**Table 1:** ERAS recommendations, reviewed by our clinicians for (a) preoperative, (b) intraoperative, and (c) postoperative care in bariatric surgery

Recommendation	Action
(a)	
Perioperative information, education, counseling	Patients and caregivers receive preoperative counseling
Prehabilitation and exercise	During the month preceding surgery patients are recommended to walk and do respiratory exercises. Any coexistent disease should be compensated and patients with CPAP ventilation for OSA (Obstructive Sleep Apnea) should be compliant to therapy for at least 4 weeks before anesthesia.
Preoperative fasting	Fluid intake up to 2 h and solid food up to 4 h prior to anesthesia induction.
(b)	
Perioperative fluid management	Goal-directed fluid therapy during surgery, the start of oral fluid intake 30 min after surgery, intravenous support only if low compliance or clinical direction
Standardized anesthetic protocol	Dexmedetomidine infusion for premedication Short-acting anesthetic agents (i.e., desforane, rocuronium) Full reversal of neuromuscular blockade (sugammadex) Opioid sparing analgesia Structured approach to airway management Bispectral Index (BIS) monitoring of anesthetic depth Monitoring of neuromuscular blockade (TOF)
PONV (postoperative nausea/vomiting)	A multimodal approach to PONV prophylaxis is adopted in all patients
Surgical approach	Laparoscopy No drains or tubes
(c)	
Early postoperative nutrition	Start fluid intake 30 min after surgery; quantitative reporting of total daily intake in the clinical record
Early postoperative mobilization	Start walking in the recovery room 30 min after surgery; quantitative reporting of total daily steps in the clinical record

Patients’ value was defined as (i) excess weight loss; (ii) better control of comorbidities; (iii) quality of life improvement; and (iv) positive experience throughout the process.

From the clinicians’ perspective value was represented by optimization of clinical parameters to get patients more fit for surgery and engaged about their own care which results in the reduction of hospital LOS and readmission rate [27, 28]. Patients’ engagement became a strategic issue to be addressed to increase the adherence to the clinical protocol and, as a consequence, to improve outcomes.

### Intervention

The IPU designed the value stream mapping (Appendix 3) both for the clinical and organizational processes (Table 2). The first contact with patients was through a dedicated reference person that gave them all the information required to be prepared for the first multidisciplinary visit. A multidisciplinary team followed patients during the whole pathway. Both individual meetings and group discussions were performed. In group meetings, structured as counseling, patients and their families shared experiences, questions, fears, and concerns with all bariatric IPU members. Patients already treated were also invited to share their bariatric surgery experience. In the preadmission phase, blood tests and clinical-instrumental examination were performed according to evidence from recent literature [29]. As an example, chest X-rays were not routinely executed, but only when a clinical condition was present. During surgery, a mini-invasive surgical approach was adopted. Early mobilization and fluid intake (30 min after surgery) granted lower pain, nausea, and vomiting.

**Table 2:** The bariatric standard process (in italics, the main changes introduced by the new process)

Pre-assessment	Assessment	Preadmission	Surgery	Recovery (30 min after awakening)	Ward	Follow-up
Dedicated contact center	Multidisciplinary (surgeon, nutritionist, psychologist)	*In a single day*	Standardized anesthesia protocol	*Early mobilization*	*A diary for self-reporting activities*	*Diary update*
*Checklist of questions and exams to be prepared for the assessment*	*Anesthesiology checklist to early detection of critical conditions*	*Multidisciplinary visit (surgeon, anesthesiologist, pain nurse)*	*Dedicated team of anesthesiologists*	*Early fluid intake*	*Mobilization and fluid intake*	*Standardized case manager follow up (1 week after surgery)*
		*Supplementary exams only if indicated after the visit*	Mini-invasive surgical approach	*Respiratory rehabilitation*	*Respiratory rehabilitation*	
		*Counseling for patients and caregivers*	*SMED and 5S of the operating room*		Pain nurse daily check	

Every patient was asked to write a diary with all the activities and symptoms experienced (drinking, walking, respiratory rehabilitation, pain, vomiting) with the aim of proactively self-monitoring the recovery progression. The nurses checked and registered all the data.

Direct contact with clinicians was guaranteed by a 24/7 unique phone number answered by one of the team members, a structured follow-up phone call was held by a case manager the week after discharge to early detect potential complications.

All data coming from these calls were collected in the electronic clinical records to allow reporting.

In January 2016, the first obese patient entered the new protocol, and we started measuring health performance. Thus, the intervention involved the following 2.122 patients until May 2018. One year after discharge patients were investigated by clinicians about their quality of life. Patients included in the one-year follow-up had surgery until June 2017.

### Outcomes

Medical data were collected in the electronic clinical records, daily updated and accessible by every professional involved using any personal computer of the hospital. A set of key performance indicators (KPIs) was developed to measure improvement:

#### Value in Patients’ Perspective

Value in Patients’ Perspective means excess weight loss (EWL) and better control of comorbidities. The percentage of EWL (%EWL) is the primary clinical outcome after bariatric surgery, leading to better control of comorbidities and return to daily activities. It could be evaluated at any time after surgery. We reported %EWL at short-term (1 year) and medium-term follow-up (3 years) to measure the sustained weight loss. The main comorbidities were as follows: hypertension, obstructive sleep apnea syndrome (OSAS), and type 2 diabetes. Bariatric surgeons and nutritionists record %EWL and recovery from comorbidities during the follow-up evaluations.

#### Quality-of-Life Improvement

Quality-of-life improvement is quantified by Patient-Reported Outcome Measures (PROMs), chosen considering the quality of life for obese patients, using the Bariatric Analysis and Reporting Outcome System (BAROS) [30]: possibility or amount of physical activities, work capability, dressing, and sexual activity were collected 1 year after surgery.

Bariatric surgeons collect PROMs during the follow-up visit 1 year after discharge.

#### Process adherence

Current literature [10–12] reported an average patients’ adherence to ERAS items at 70%, ranging from 60% to more than 80%, depending on patients’ age and the clinical phase. A relevant issue is to increase the adherence to protocol until a complete observance, considering its effect on survival [13] and outcomes [12]. We investigated the effect of patients’ engagement and commitment on adherence with a consistent data collection. We analyzed the percentage of patients attending: (i) activities for motivation and preparation (counseling, prehabilitation, and personal diary’s drawing up); (ii) ERAS protocol, including early mobilization and fluid intake in the postoperative phase to reduce pain and clinical complications; (iii) follow-up phone call and visit 1 year after surgery to grant the sustainability of the achieved results.

LOS was measured as a primary output of process’ adherence.

#### Clinical Outcomes

Clinical outcomes are as follows: mortality, morbidity, readmission and reoperation rates within 30 days after surgery; mortality 1 year after discharge; postoperative pain, nausea and vomiting in day zero, one and two after surgery and 1 week later. Access to our local data warehouse allowed direct comparison of data sets for each unit (i.e., bariatric surgery unit) with regional and national registers (i.e., AGENAS, National Agency for Regional Health Services).

#### Resources, Value, and Costs

Cost control department measured the direct resources relate to the cost for the clinical treatment and the freed-up resources due to process optimization in bariatric surgery, available for other patients in the hospital. All the analyses related to resource consumption were registered in our data warehouse and used daily.

### Patient Involvement in the Research Project

Patients and their families partnered with us during the whole process. At the beginning patients undergoing standard care were asked for feedbacks and suggestions to improve, using structured interviews.

During the implementation of the intervention, patients actively participated in their own care and worked on items with their care-givers.

To grant results’ sustainability, periodic meetings with previous patients or patients to be and their caregivers were organized to keep them motivated, sharing their experience and having new proposals to improve.

This original contribution was a single-center observational cohort study of elective bariatric surgical patients. It aimed to implement the VBHC approach on ERAS protocol and to measure the impact of the engagement of patients and a multiprofessional team on outcomes, quality of life and costs, to prove its feasibility and playable.

## Results

### Studied Patients

From January 2016 to May 2018, we studied 2.122 morbidly obese patients (MOP), of whom 89% underwent sleeve gastrectomy and 11% gastric bypass surgery.

The demographic characteristics of the patients are shown in Table 3.

**Table 3:** Demographics of the studied patients

Male	30%
Female	70%
Age (years)	42 ± 11
BMI	45 ± 6.6
Hypertension	65%
Obstructive sleep apnea syndrome	15%
Type 2 diabetes	7.8%

### Outcome

#### Value for Patients

##### Percentage Excess Weight Loss

The %EWL measured at the short-term follow-up after surgery was 74.05%, consistently higher than the European average (58.49%) registered in the database of the European Accreditation Council for Bariatric Surgery (EAC-BS).

The medium-term follow-up data, 3 years after surgery, further improved the obtained results. The %EWL was 82.03%, steadily higher than the European average (51.89%) (source: EAC-BS).

##### Recovery from Comorbidities

**Fig 1:**
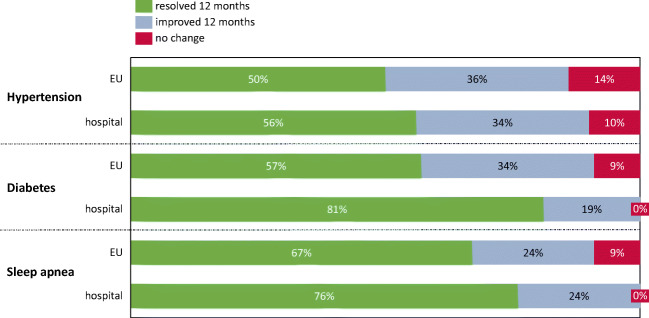
MOP comorbidities: comorbidities resolved (no drugs any more), improved (reducing drugs’ number or dose), or without change after 12 months for our patients compared with those of European data

Together with %EWL, improvement of other clinical conditions and comorbidities was investigated 1 year after surgery and compared with those of other European countries. Considerable results were obtained, as shown in Fig. 1. Patients, for the most part, recovered from comorbidities, thus, discontinuing or reducing pharmacological treatment. The most consistent result was on type 2 diabetes: 81% of patients discontinued the therapy, obtaining normal values of glycathed hemoglobin (HbA1c).

##### Quality-of-Life Improvement as PROMs

One year after surgery patients underwent an interview assessing quality-of-life parameters. Seventy-seven percent of the patients reported to work better and more than before the procedure. Eighty-nine percent were able to practice physical activities, and 52% reported a longer training time. Ninety-two percent of patients were buying clothes everywhere and not only in special shops for oversized customers. Ninety percent graded their sexual life as “good” and 48% reported an improvement.

##### Process Adherence

ERAS recommendations (Table 1) were applied and compliance was monitored by physicians using a pre-operative questionnaire. All the patients attended counseling, prehabilitation, and exercises. Regarding pre-operative tests, the process optimization resulted in waste reduction and patients spent 40% less time at the hospital completing all the exams in a single morning.

Ninety-two percent of them had oral fluid intake up to 2–8 h before surgery. Preoperative fluid management and PONV prophylaxis were adopted in 100% of patients. Regarding anesthesia, a standardized anesthetic protocol was fully applied. All the surgical procedures were performed using laparoscopy and in 98% of the cases, no drains or tubes were placed.

Postoperative intensive care admittance was almost zero (0.2%), mainly because of tight clinical management of all the comorbidities before surgery (i.e., CPAP for OSAS, glycemic control, hypertension control).

All patients received support to start oral fluid intake and mobilization 30 min after surgery. Moreover, 98% of them recorded their progress in a diary.

This standardized patient-centered approach resulted in an average LOS, of 2.1 days for all the 2.122 patients. Responders of follow-up phone calls from the case manager were 82%, 7 days after surgery and compliance to the 1-year follow-up visit with the surgeon was 83.4%.

##### Clinical Outcomes

Retrospective data analysis from the studied population revealed zero mortality and 1.8% overall morbidity within 30 days of surgery. Mortality was zero 1 year after discharge. Both readmission and reoperation rates were 0.4% within 30 days of surgery.

During the whole hospitalization, 77.5% of MOP experienced no pain at all (NRS < 3). 22.5% of the patients reported pain or discomfort and were successfully treated with rescue therapy as protocol. Regarding Post Operative Nausea and Vomiting (PONV), 28% reported mild nausea, 11% vomiting, 61% reported no symptoms. One week after discharge the prevalence of nausea and vomiting was 6%.

##### Costs

Table 4 summarizes the clinical changes responsible for the main impact on resources, the additional costs and freed-up resources. While the new process entails additional direct resources for counseling for every patient, it frees up time on chest X-ray machine which could offer additional chest X-rays to other patients. Because of the new anesthetic protocol and the early post-surgical mobilization (30 min after surgery), patients had better pain and vomiting control, leading in turn to 40% lower drug prescriptions.

**Table 4:** Main clinical changes that impact on resources

	Previous protocol	New intervention	Additional costs (*) in €	Freed-up resources (*) in €
Counseling and prehabilitation	None	All patients	100	
Chest X-ray	All patients	Only if needed due to specific clinical condition		−53.3
ERAS recommendations	Intraoperative applied	fully applied		−9.9
Postoperative intensive care unit usage	Scheduled for some patients	None scheduled		−38.5
			100	−102

*Average for patient

Given the financial value of resources involved, additional costs associated with counseling are compensated by the additional revenues obtained from the freed-up chest X-rays, no ICU utilization, and lower drug usage.

## Discussion

Our intervention confirms the positive impact of bariatric surgery on clinical outcomes and significant improvement of quality of life for MOP. The care of these patients requires much more than an effort on clinical and technical skills only. Understanding how patients and their families’ motivation, education, and involvement can impact their outcomes has been the key to implement the cultural change needed to achieve better overall results. We redesigned the bariatric process taking into deep consideration all the milestones mentioned above starting from an evidence-based clinical pathway (according to ERAS principles [9] shaped on MOP needs as shown in Table 1 and Nice Guidelines [29]) and international benchmarks [31–33], to put the basis of a standardized model of care integrated with a structured motivational and educational support.

The turning point to reach this goal has been redesigning the process of adopting a multidisciplinary approach and considering the different points of view of entities involved: (i) patients and families; (ii) clinicians; and (iii) cost and asset management and control.

Translating clinical and patients’ needs in organizational changes has been possible placing patient value first.

From a methodological point of view, we created a framework to maximize the value coming from a multidisciplinary inclusive approach and we invested in patients’ engagement to obtain full adherence (from 82% to 100%), leading to better clinical and patient-oriented outcomes. Also, we measured costs of the full cycle of care as the VBHC approach suggests (Appendix 1).

Engaging patients and caregivers, we experimented full compliance with key clinical steps: all patients were able to drink and walk 30 min after surgery in the recovery room and we recorded less request of analgesic and antiemetic drugs, likely as a direct consequence of the early movement. Nurses accompany patients on their first excursions and, progressively, as they begin to show confidence and a positive attitude, they can walk with their familiars or friends along with the ward. Self-reporting activities on a diary keep patients motivated and aware of their progresses, which contributes significantly to compliance. Consistent evidence already supported the positive impact on outcomes from patients and caregivers motivation, education, and direct involvement in their own pathway of care [34, 35].

Twenty percentage points of patients’ adherence higher than previous studies confirmed the relevance to add to a patient-centered care approach (ERAS) a patient wellness framework (VBHC on top of ERAS). The core is the care relationship between the patient and the multidisciplinary team.

Other research studies showed the positive impact of VBHC on quality and costs, even in complex surgical systems [24] but without exploring the important impact on the quality of life for patients.

For the first time, PROMs have been measured together with clinical outcomes in our hospital. Even if PROMs are currently being debated, there is a global consensus on their relevance with traditional measures of healthcare to improve clinical care [36–38]. Consistent evidence suggests obese employees could only reach approximately 80% of the productivity of a normal-weight worker [2, 39]. One year after surgery, 77% of our patients reported to work better and more than before the intervention. The above-stated issue was strictly related to better performance in physical activities and a positive approach to daily life.

In our opinion, based on the overall results (clinical, PROMs, and adherence), patients’ and caregivers’ involvement with the IPU personnel since the beginning, through counseling, multidisciplinary approach, and close follow-up, have been the key elements of success and the real innovation in our organization.

We measured the economic impact of the new pathway and it is sustainable because every economic saving (i.e., no ICU utilization and lower drug usage) is invested in quality and activities to involve patients (i.e., counseling).

Communication among IPU members, patients, and board management staff has been crucial during this process.

Dedicated meetings were organized to share results and progress.

To our knowledge, this is the first VBHC study implemented in Italy. We are convinced that this could be a good example of patients’ and families’ engagement in since they ultimately became part of the team.

The measurement of impact on social costs is the next challenge and a further step for an even larger improvement.

The Value-Based Model developed from bariatric surgery experience has become the prototype for redesigning other surgical pathways in our hospital (breast cancer, esophagus, and prostate cancer), and the aim is to be a case study for other organizations to set this approach.

We studied and implemented the new pathway for all MOP, and its limit is that it is not a randomized controlled trial. Having worked with already elaborated results from databases of clinical records, we recognize as limitation of our study the lack of a detailed analytic approach based on single-patient data.

## Conclusion

This methodological and descriptive report shows the feasibility of applying Value-Based Healthcare and ERAS to obtain patients’ engagement in the full cycle of care.

Building a caring relationship by a multidisciplinary team, adding patient wellness in a VBHC framework on top of ERAS as a patient-centered approach, increases patients’ adherence to the pathway of care, resulting in better health outcomes (clinical and PROMs).

It is sustainable: all the savings were invested in activities for patients’ motivation.

It is replicable: other surgical pathways in our hospital have been redesigned using the bariatric model.

## Appendix 1. Value-Based Healthcare

It has been developed by M. Porter and E. Teisberg [14] to delivering superior patient value, measuring value as patient health outcomes per dollar spent.

It is based on 6 simple steps:

1. Organize care into integrated practice units (IPUs) around patient medical conditions
2. Measure outcomes and costs for every patient
3. Move to bundled payments for care cycles
4. Integrate care delivery systems
5. Expand geographic reach
6. Build an enabling information technology platform

In VBHC, the multiprofessional team is crucial, including patients and their families with an approach “patient center, doctor driven”, as well as international collaboration, cost-effectiveness within the full cycle of care and the development of models having potential for upscaling.

In VBHC, it is essential to incrementally improve initiatives and scale-up to attract enough patient volume.

## Appendix 2. Lean Principles

The term Lean was coined by J.P. Womack and D.T. Jones to describe the Toyota Production System that managed to get by the half of space, labor effort, capital, inventory, and far fewer than half the defect and safety incidents [40]. Lean is more than a toolset; it is a management system for continuous improvement and for employee engagement that allows to create problem solvers [41].

It is based on 5 principles [42]:

Even if lean was born in the automotive industry, every type of organization, including healthcare, has to face customer and employee satisfaction, cash flow, and quality.

Lean methodology moved from manufactory to healthcare in 2001, with Virginia Mason Institute, Seattle Children’s Hospital, and Royal Bolton Hospitals as pioneers. Our hospital started the lean journey in 2011, and we are close to the 5 principle interpretation for healthcare suggested by Intermountain Healthcare [43], inspired by Spear and Bowen [44].

1. Every change must move the organization closer to this ideal, meaning:

- Exactly what the patient needs, defect-free
- One by one, customized to each patient
- On demand, exactly as requested
- Immediate response to problems or changes
- No waste
- Safe for patients, staff, and clinicians: physically, emotionally, and professionally

2. Problems are quickly identified because not conform to the ideal. Employees are the experts and the organization must help them to become problem solvers, in the recognition that employees are assets rather than expensive items.
3. The process must change as soon as a better way is known. Countermeasures are implemented as scientific experiments and results are measured.
4. Processes improve when connections are direct, simple, and binary—doing so makes them prompt, efficient, and reliable.
5. These simple rules not only define the ideal work process but enable workers to quickly see opportunities to improve.

## Appendix 3. Value Stream Map [43]

VSMs visually show the key people, material, and information flows required to deliver a product or service. It is used to understand the high-level view of a process to distinguish value-added and nonvalue-added activities. As an example in healthcare, a value-added activity is the doctor’s interview to obtain clinical information, whereas nonvalue-added activity is the waiting time.

Once a problem is recognized, specific problem solving can be focused to improve the process using lean tools.

As a consequence, a “future state map” is created that outlines a clear goal toward which the team applies its improvement efforts.
